# Application of a combined approach including contamination indexes, geographic information system and multivariate statistical models in levels, distribution and sources study of metals in soils in Northern China

**DOI:** 10.1371/journal.pone.0190906

**Published:** 2018-02-23

**Authors:** Kuixian Huang, Xingzhang Luo, Zheng Zheng

**Affiliations:** Department of Environmental Science & Engineering, Fudan University, Shanghai, China; Nankai University, CHINA

## Abstract

The purpose of this study is to recognize the contamination characteristics of trace metals in soils and apportion their potential sources in Northern China to provide a scientific basis for basic of soil environment management and pollution control. The data set of metals for 12 elements in surface soil samples was collected. The enrichment factor and geoaccumulation index were used to identify the general geochemical characteristics of trace metals in soils. The UNMIX and positive matrix factorizations (PMF) models were comparatively applied to apportion their potential sources. Furthermore, geostatistical tools were used to study the spatial distribution of pollution characteristics and to identify the affected regions of sources that were derived from apportionment models. The soils were contaminated by Cd, Hg, Pb and Zn to varying degree. Industrial activities, agricultural activities and natural sources were identified as the potential sources determining the contents of trace metals in soils with contributions of 24.8%–24.9%, 33.3%–37.2% and 38.0%–41.8%, respectively. The slightly different results obtained from UNMIX and PMF might be caused by the estimations of uncertainty and different algorithms within the models.

## Introduction

It is well known that heavy metals in soils present risks for human health due to their toxicity, persistence, and non-biodegradable natures [1]. Urban and industrial areas are generally considered a sink of trace metals from various pollution sources [2]. In China, vehicular emissions, industrial waste, and atmospheric deposition are the most important sources of heavy metals in urban topsoil, whereas wastewater irrigation and land fertilization with sludge contribute most to heavy metal contamination of topsoil in rural areas [3]. Due China’s rapid urbanization and industrialization, the establishment of industrial operations and fast urban expansion have drastically increased industrial and municipal wastewater discharges and other pollutant emissions nationwide [4]. Consequently, strengthening the prevention and control of soil pollution has become China's current key strategic task [4].

Tianjin City, located in Northern China, is an important industrial base. Since the 1980s, Tianjin has undergone rapid agricultural and industrial development, so heavy metals are more or less constantly emitted into the soil via atmospheric deposition, solid waste emissions, application of pesticides and fertilizers and wastewater irrigation from sewage and industrial effluents [5]. In particular, wastewater irrigation for agriculture has been used for nearly 50 years, to some extent, leading to the accumulation of metals in soils [6]. Studies have found a significant enrichment of trace metals in the topsoil of Tianjin [7]. Elevated soil trace metal concentrations in Tianjin have attracted attention from both the government and the public. The Work Plan of Soil Pollution Prevention and Control of Tianjin was published by the Tianjin Municipal Government in January 2017 [8]. Therefore, understanding the contamination characteristics of trace metals in soils and identifying their potential sources are important for developing appropriate pollution prevention and control regulations [9, 10]. However, few reports have focused on the source apportionment of trace metals in the soils of Tianjin.

Source apportionment of heavy metals in soil uses quantitative methods to accurately identify natural and anthropogenic sources and apportion their contribution [10]. The receptor model is a general approach for apportioning the contribution from all major sources based on the concentration profiles observed at sampling sites [10, 11]. Several receptor models, such as the chemical mass balance (CMB) model, principal component analysis/absolute principal component scores (PCA/APCS), APCS/multiple linear regression, positive matrix factorization (PMF), and UNMIX, have been proposed over the past several decades [12]. Although these models have their own characteristics and limitations, they have been widely used and have shown good applicability in source apportionment of pollutants in various environmental media, including the atmosphere, sediments, and water.

Among them, the UNMIX and PMF models can apportion potential sources without prior knowledge of source profiles, and they incorporate non-negative constraints into their calculation procedures, which makes their results more interpretable [10]. In addition, the UNMIX and PMF models have been recommended by the United States Environmental Protection Agency (U.S. EPA) as general modeling tools for source apportionment studies [10, 12]. However, the source apportionment of soil trace metals in large-scale regions is challenging because of the high spatial variability of heavy metal contents in topsoil that is caused by both heterogeneous parent materials and widespread human activities. In these cases, the spatial variability of the trace metal concentrations in soils is basic information for identifying the possible sources of contamination. Mapping based on geographical information systems (GIS) and spatial analysis is widely used to understand spatial distribution patterns and identify the likely sources of metals in surface soils. Therefore, receptor models combined with GIS may be an effective method that can improve the accuracy of source apportionment of soil trace metals.

In this study, a detailed investigation was conducted to comprehensively understand the levels and spatial distribution in soils of the Tianjin area and to identify their potential sources. Geochemical methods, including the geoaccumulation index and enrichment factor, were used to analyze the contamination characteristics of trace metals in soils. Receptor models, including the UNMIX and PMF models, were applied to identify trace metal sources and calculate source contributions. The systematic research presented here provides significant information for understanding the applications and challenges of PMF and UNMIX in the source apportionment of soil heavy metals at regional scale. In addition, the results will be useful and helpful for managers of soil environments to select and implement suitable measures for soil pollution prevention and control in Tianjin.

## Materials and methods

### Study area

Tianjin (N: 38°34′–40°15′; E: 116°43′–118°04′), in the lower reaches of the Haihe River System, is an important industrial metropolitan area in northern China. Major industries include metal smelting and pressing, machine manufacturing, petrochemical industry, electronic equipment manufacturing, automotive manufacturing, chemical industry, pharmaceutical manufacturing and production of electric and heat power; there are several other industrial activities [13–15]. Tianjin covers a total area of 11,900 km^2^ and had a population of 12.99 million in 2010 [13]. This region, close to Bohai Bay, which is affected by the ocean climate and has a semi-humid monsoon climate, that is characterized by wide seasonal variation in annual rainfall of approximately 600 mm [13]. The landform of its north is higher in elevation than is that in the south [13]. The altitude ranges from 3.5 to 1052 m [13]. The main soil types are endoaquept, haplaguept and hapludalf [13].

### Sample collection and analysis

A total of 171 surface soil samples (0–20 cm) were collected in May 2013, which reasonably represent soils of the entire study area. Sample collection and preparation, analytical methods and quality control were conducted according to the Technical Specification for Soil Environmental Monitoring of China [16].

Before field work, sampling coding system and sampling cell were made on a 1:250,000 digital maps in national fundamental geographic information system. The size of sampling grid (sampling density) varied with land use patterns, with an 8 km×8 km grid for cultivated land, a 16 km×16 km grid for forested land and grasslands, and a 40 km×40 km grid for unutilized land. Each total sample was a composite material taken from several sub-sample (0–20 cm) over a 50 m × 50 m patch of land. Ten to thirty sub-samples were collected at sites that did not have a not flat landscape and had heterogeneity in soils by using the S-shaped method. Five to nine sub-samples were collected at sites in cropland by using diagonal method. Five sub-samples were collected at sites with flat landscapes and homogeneous soil using the quincunx method. Samples for measuring organic compounds were collected with a pre-cleaned stainless steel scoop in a pre-cleaned brown glass bottle with a Teflon cap and were immediately transported to the laboratory and stored at -4°C until analysis.

Soil sample preparation for element analysis was performed as follows [17]. Each soil sample (10–20 mg) was digested in 1 mL of 60% (w/w) HNO3 and 1 mL of 60% (w/w) HClO4 in a stainless steel high-pressure digestion bomb at 140°C for 6 h. After completely cooling the system, the open vial was transferred to a hot plate (about 190°C) to evaporate the solution until the volume had decreased to several hundred micro-liters, then 0.5 mL of 49.5% (w/w) HF was added and the sample was evaporated again. The HF treatment was repeated several times until the silicate minerals had been completely dissolved. Finally, the residual solution was diluted to 6 mL with 1% (w/w) HNO3, filtered through a syringe filter (0.45 μm). The total concentrations of As, Hg and Se were analyzed using atomic fluorescence spectrometry (AFS); the total concentrations of the other elements were determined using inductively coupled plasma mass spectrometry (ICP-MS). The detection limits were 0.2, 0.1, 0.7, 5.0, 0.5, 0.005, 5.0, 0.5, 0.5, 5, 1.0 and 5.0 mg kg-1, for As, Cd, Co, Cr, Cu, Hg, Mn, Ni, Pb, V, Se and Zn, respectively. Quality assurance and quality control procedures were performed in conjunction with laboratory analyses by analyzing standard reference materials GSS-1, GSS-2, GSS-3, and GSS-4 soil (National Research Center for Geoanalysis of China). The results showed that the precision and bias of the analysis were generally below 5%. Recoveries of samples spiked with standards ranged from 95 to 105%.

### Geochemical methods

#### Enrichment factor

An enrichment factor (EF) is a commonly used geochemical criterion to evaluate the degree of heavy metal pollution [18]. It is defined as follows:

$$EF=(\frac{C_{n}}{C_{ref}}{)}_{sample}/(\frac{B_{n}}{B_{ref}}{)}_{background}$$ (1)

where (C_*n*_/C_*ref*_)_*sample*_ is the concentration ratio of the examined metal and the reference element in soil samples, and (B_*n*_/B_*ref*_)_*background*_ is the natural background value of the examined metal to the reference element ratio. The elements that are most often employed as reference elements include several conservative elements, such as Sc, Mn, Al, and Fe. [19]. In this study, Mn was used as the reference element. The background values of soil trace metals in the Tianjin area (CNEMC 1990) were used for calculating the EF values. The five-category system proposed by Sutherland [20] was used to classify samples: no or minimal enrichment (EF<2), moderate enrichment (2≤EF<5), significant enrichment (5≤EF<20), very high enrichment (20≤EF<40), and extremely high enrichment (EF≥40).

#### Geoaccumulation index

The geoaccumulation index (I_*geo*_), was first proposed by Müller [21] to assess the pollution levels of bottom sediments, and has been widely applied to the assessment of contamination by trace metals in soil. It is calculated as follows:

$$I_{geo}=\log_{2}(C_{n}/1.5B_{n})$$ (2)

where C_*n*_ is the concentration of the examined trace element (*n*) in the soil samples, and B_*n*_ is the background value for the corresponding trace element. A constant of 1.5 is used to compensate for possible variations in the background data because of lithological variations as well as very small anthropogenic influences [19]. I_*geo*_ was calculated using the background values in order to compare the calculation results of EF and I_*geo*_. The I_*geo*_ consists of 7 classes [18]: Class 0 (I_*geo*_≤0), uncontaminated; Class 1 (0<I_*geo*_≤1), uncontaminated to moderately contaminated; Class 2 (1<I_*geo*_≤2), moderately contaminated; Class 3 (2<I_*geo*_≤3), moderately to heavily contaminated; Class 4 (3<I_*geo*_≤4), heavily contaminated; Class 5 (4<I_*geo*_≤5), heavily to very heavily contaminated; Class 6 (I_*geo*_≥5), very heavily contaminated.

### Source apportionment models

Both UNMIX and PMF are advanced multivariate receptor models that are based on factor analysis, and constrain all of the elements in the factorized matrices to nonnegative. They determine the number of sources as well as their chemical compositions and contributions without the source profile data. In the models, the sample data matrix is decomposed into two matrices (factor contributions and factor profiles), with a residual matrix [5, 22]. The matrix equation can be expressed as:

$$x_{ij}=\sum_{k=1}^{p}g_{ik}f_{kj}+e_{ij}$$ (3)

where *x*_*ij*_ is the *j*th measured element concentration in the *i*th sample, *p* is the number of factors that contribute to the samples, *g*_*ik*_ is the relevant contribution of factor *k* to the *i*th sample, *f*_*kj*_ is the concentration of element *j* in factor *k* profile, and *e*_*ij*_ is the residual error matrix. Details of the two models are described in the following sub-sections.

#### UNMIX

The UNMIX program includes two key algorithms, that are linked by the matrix operation of Singular Value Decomposition (SVD). Given a data matrix with *n* samples and *m* species, the first step is to perform the SVD to reduce the dimensionality of the data space from *m* to *p*, the number of sources. The number of sources (*p*) is estimated by the NUMFACE algorithm based on signal-to-noise considerations [23]. The next algorithm is to find data "edges" (more generally, hyperplanes) in a (*p*−1)-dimensional space, which are then used to determine the compositions and contributions of the *p* sources [24]. It should be noted that UNMIX retains only the species that contribute to the improvement of the model's signal-to-noise ratio. In this work, U.S. EPA UNMIX version 6.0 was used.

#### PMF

Compared to UNMIX, the PMF model considers the uncertainty of each data point individually and is built on a completely different algorithm [25, 26]. The core of the PMF algorithm is to minimize the object function Q in the following way [27]:

$$Q=\sum_{i=1}^{n}\sum_{j=1}^{m}(\frac{e_{ij}}{\mu_{ij}}{)}^{2}$$ (4)

where *μ*_*ij*_ is the uncertainty of the *j*th species concentration for the *i*th sample, *n* is the number of samples, and *m* is the number of species.

In this study, U.S. EPA PMF version 5.0 was employed based on the multilinear engine-2 (ME-2) algorithm. ME-2 solves the PMF equation iteratively using the conjugate gradient algorithm, minimizing the Q function [28]. The model was used in robust mode to avoid the impact of outliers on the PMF model results. Additionally, the application of the PMF model depends on the uncertainty of each data point to reduce the influence of noise in the environment data. In this study, for data above the method detection limit (MDL), the uncertainty matrix (*μ*_*ij*_) was calculated to be 10% of *x*_*ij*_ plus MDL divided by three (*μ*_*ij*_ = 0.1 × *x*_*ij*_ + MDL/3). If *x*_*ij*_ is below or equal to MDL, *x*_*ij*_ values below MDL were replaced with MDL/2, and the uncertainty matrix is estimated to be 20% of *x*_*ij*_ plus MDL divided by three (*μ*_*ij*_ = 0.2 × *x*_*ij*_ + MDL/3) [29].

### Geostatistical tools

Kriging and inverse distance weighting (IDW) are the two most commonly used geostatistical interpolation methods for characterizing spatial patterns of soil contamination. Kriging is a linear interpolation technique that exhibits the best linear unbiased estimates for spatial variables [30]. However, the relatively easy method of IDW is more appropriate for the purposes of this study of assessing the spatial variations of soil trace metals contamination based on EF and I_*geo*_ values and for identifying the affected regions of pollution sources obtained from UNMIX and PMF models. It uses a specific number of nearest points, which are then weighted according to their distance from the point being interpolated [31]. The IDW interpolation maps were produced using ArcGIS 10.2 software.

## Results and discussion

### Contamination characteristics

The soil pH values varied from 5.9 to 9.0 with a mean value of 8.24. Because 93.6% of soil pH levels were above 7.5, this indicated that most soil of this region is alkaline soil. The basic statistics for the concentrations of measured trace metals in surface soils from the Tianjin area are listed in Table 1. From Table 1, it apparent that only that the median and mean concentrations of only Cd, Pb and Zn were higher than their corresponding average background values (ABVs). Approximately 76.6% of Cd samples, 74.3% of Pb samples and 54.4% of Zn samples exceed their corresponding ABVs. In the Chinese soil quality guidelines [32], Grade I represents the average natural levels for uncontaminated soil and Grade II indicates that intervention should occur to protect human health [33]. Compared with the Chinese soil quality guidelines [32], 24.6%, 15.8%, 13.5%, 15.2%, 32.8% of the Cd, Cu, Hg, Ni and Zn concentrations exceed their corresponding Grade I values. Additionally, the highest concentrations of these five trace metals even exceeded their Grade II values. It was also found that the mean concentrations of most trace metals, except Co and V, were lower than the target values that are recommended by in the Netherlands soil contamination guidelines [34].

**Table 1:** Summary statistics of trace metal concentrations in soil samples and some reference values (mg kg^-1^).

Trace metal	As	Cd	Co	Cr	Cu	Hg	Mn	Ni	Pb	Se	V	Zn
Minimum	0.67	0.010	3.7	10.0	10.4	0.004	285	12.1	8.6	0.016	10.4	12.8
50th	9.17	0.150	13.2	66.5	25.0	0.037	615	30.8	25.8	0.145	77.0	82.5
Mean	9.34	0.183	13.3	67.9	28.1	0.076	681	31.4	26.4	0.149	76.8	101.0
Maximum	20.50	1.310	26.2	200.0	110.0	0.755	1720	68.5	74.0	0.734	148.0	406.0
Standard deviation	3.86	0.141	4.1	21.7	14.1	0.109	258	10.0	7.6	0.089	28.1	61.0
Coefficient of variation	0.4	0.8	0.3	0.3	0.5	1.4	0.4	0.3	0.3	0.6	0.4	0.6
Skewness	0.37	4.26	0.49	1.50	2.68	3.54	1.28	0.93	1.66	2.98	0.04	2.15
Kurtosis	0.23	26.82	0.37	8.76	10.25	16.04	2.28	1.74	8.32	16.86	-0.16	5.93
Average background of Tianjin	9.60	0.090	13.6	84.20	28.800	0.084	686.00	33.300	21	0.18	85.200	79.3
C-Grade I	15	0.2	-	90	35	0.15	-	40	35	-	-	100
C-Grade II	30.0	0.6	-	200.0	100.0	0.5	-	50.0	300	-	-	250
D-Target	29	0.8	9	100	36	0.3	-	35	85	0.7	42	140

C-Grade I: Grade I of Chinese soil guidelines; C-Grade II of Chinese soil guidelines; D-Target: Target values of Dutch soil guidelines.

**Fig 1:**
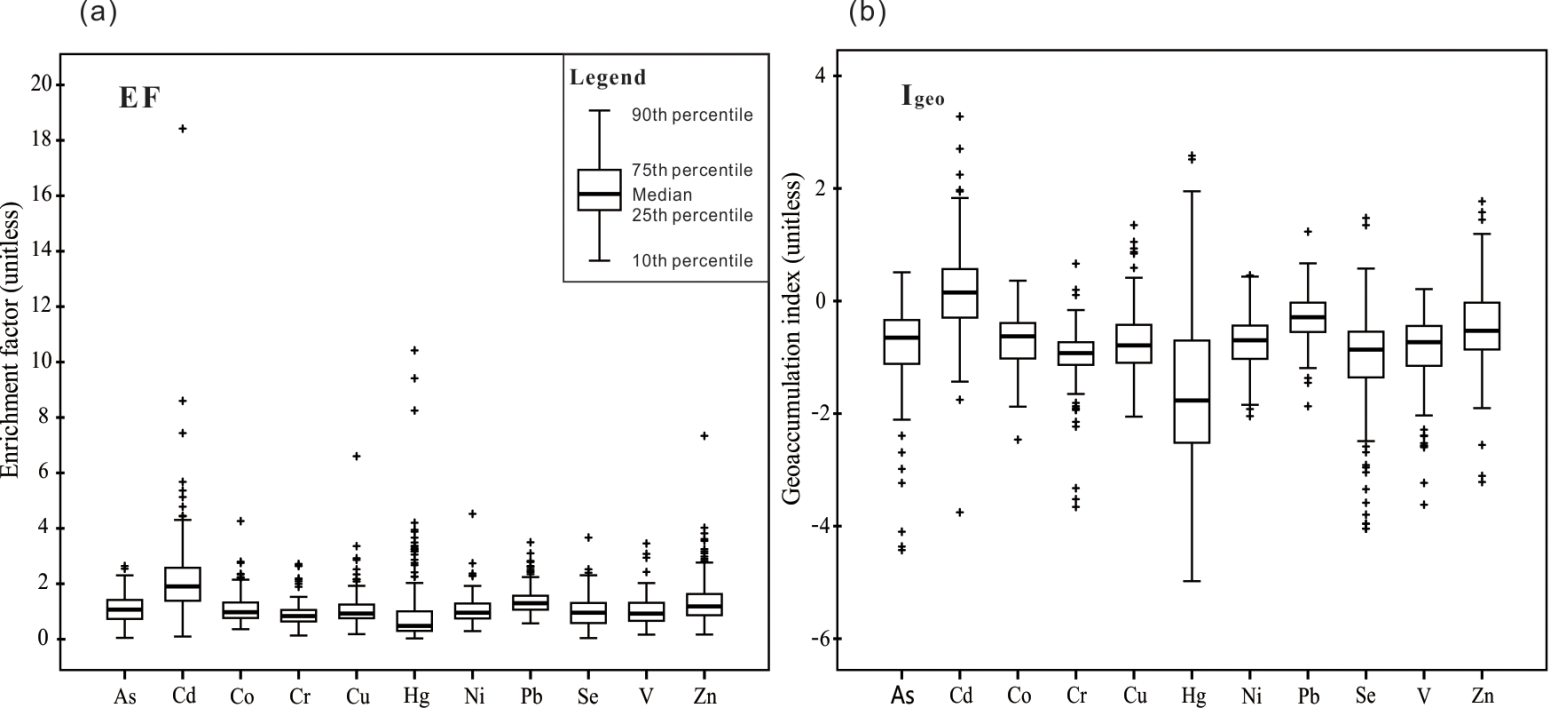
Boxplots of enrichment fator (EF) and geoaccumulation index (Igeo) for trace metals in soil samples.

To evaluate the extent of trace metals contamination and anthropogenic inputs, EF and I_*geo*_ values of different elements were calculated separately for all sampling sites (Fig 1; Table 2; S1 Table). EF values showed that Cd was the most serious metal contaminant in soils, with 47.9% of samples classified above the moderate contamination level (Fig 1(A); Table 2(A)). Both Pb and Zn had the same average EF of 1.38, with 16.2% of samples for Pb and 22.0% of samples for Zn with EF >2. Although the mean EF value for Hg was below zero, 17.1% of EF values for Hg were in the range of 2–5 and 2.6% of EF values were in the range of 5–20, that influences Hg was also an important pollutant influencing soil quality in Tianjin. At least 93% of EF values of the other trace metals showed no or minimal contamination, with only a few individual sampling sites with EF values >2.

**Table 2:** Percentages of class distribution for pollution assessment of trace metals in soil samples using enrichment factor index (a) and geoaccumulation index (b) (n = 171; %).

Trace metals	As	Cd	Co	Cr	Cu	Hg	Ni	Pb	Se	V	Zn
(a)											
no or minimal enrichment	95.3	52.1	93.0	96.5	94.7	80.3	97.1	83.8	95.3	94.9	78.0
moderate enrichment	4.7	44.4	6.4	3.5	4.7	17.1	2.9	16.2	4.7	5.1	21.4
significant enrichment	0.0	3.5	0.6	0.0	0.6	2.6	0.0	0.0	0.0	0.0	0.6
(b)											
Uncontaminated	89.5	41.5	94.7	98.3	91.8	85.4	95.3	77.2	97.1	95.9	74.9
uncontaminated to moderately contaminated	10.5	45.6	5.3	1.8	7.0	8.8	4.7	22.2	1.8	4.1	19.9
moderately contaminated	0.0	11.1	0.0	0.0	1.2	4.7	0.0	0.6	1.2	0.0	5.3
moderately to heavily contamined	0.0	1.2	0.0	0.0	0.0	1.2	0.0	0.0	0.0	0.0	0.0
heavily contaminated	0.0	0.6	0.0	0.0	0.0	0.0	0.0	0.0	0.0	0.0	0.0

**Fig 2:**
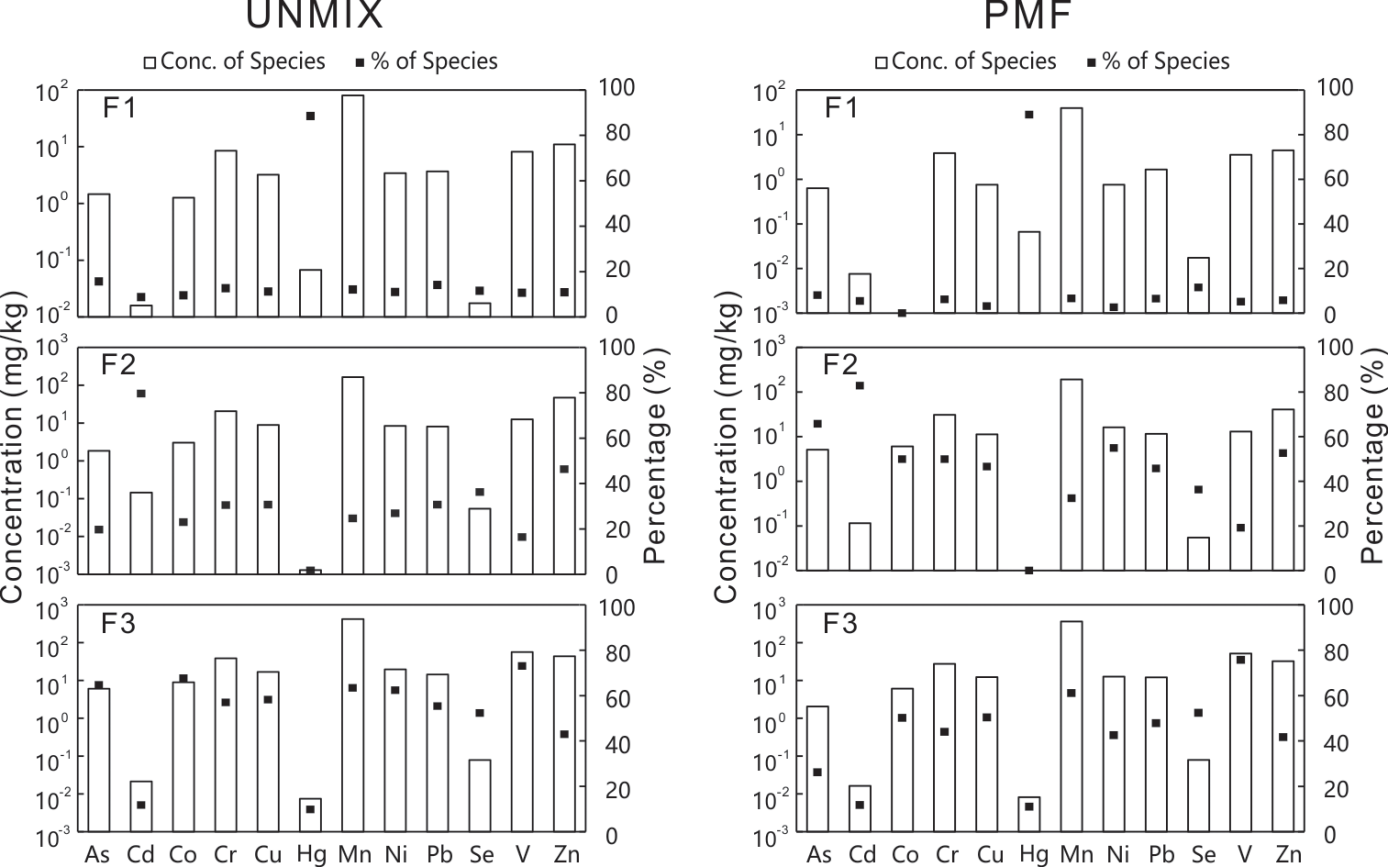
Concentration (mg/kg) and corresponding percentage (%) of the species in the different factors obtained by Unmix (Left) and PMF (Right).

The mean I_*geo*_ values for these trace metals, except for Cd (mean I_*geo*_ = 0.19), were lower than 0, indicating no soil contamination. The I_*geo*_ values of Cd varied the most, ranging from uncontaminated to heavily contaminated. The proportions of these elements in soil samples with I_*geo*_ > 0 decreased in the following order: Cd (58.5%) > Zn (25.1%) > Pb (22.8%) > Hg (14.6%) > As (10.5%) > Cu (8.2%) > Co (5.3%) > Ni (4.7%) > V (4.1%) >Se (3.0%) > Cr (1.8%) (Fig 2(B), Table 2(B)). The maps of EF and I_*geo*_ for these elements exhibited very similar spatial distributions, confirming the interpolation results (S2 Fig). The classification of samples by EF was more severe than that by I_*geo*_, which was consistent with the report by Chabukdhara and Nema [35]. Overall, Cd, Hg, Pb and Zn showed the relatively high contamination levels. In particular, Cd was the most significant pollutant in Tianjin soils.

According to Zhang and Liu [36], EF values >1.5 suggeste that the metal was derived mainly from anthropogenic sources. From S1 Fig, the estimated maps of the spatial distribution of trace metal EF values showed that the soils in different districts of Tianjin were affected by human activities to varying degrees. Cd contamination was observed over a wide area, which might be associated with agricultural activities, such as fertilizer use and sewage irrigation. Although Pb and Zn showed similar spatial distributions, the hot-spot areas for Zn were wider than those for Pb, which mainly distributed in the suburban and the northern areas of Tianjin. The high EF values of Hg mainly occurred surrounding these districts that cross the Dagu Ditch and the Ji district in the north of Tianjin, demonstrating obvious regional characteristics. The Dagu Wastewater Drainage River, located in the south of Haihe River, is an important urban drainage channel that has received municipal sewage and industrial waste water for a long period of time [37]. The main industries that types draining waste into this channel include electroplated metal products industry, chlor-alkali industry, paper manufacturing industry, the leather industry, and textile industry [38]. Previous studies have noted that Hg in agricultural soils near the Dagu Ditch was one of the most serious pollutants because the Dagu wastewater is used for irrigation [39]. The high EF values for As, Co, Cr, Cu, Ni, Se and V were distributed only sporadically in the study area, indicating that these seven elements were less influenced by anthropogenic emissions.

### Comparison of source profiles

To apportion the sources of soil trace metals in the Tianjin area, both the UNMIX and PMF models were comparatively applied. First, the optimum number of sources needs to be determined when running these two models. Generally, we can use the Min. R^2^ value and Min S/N value as the diagnostic basis for the UNMIX model. Here, Min. R^2^ means the minimal explained variance of each species under the specified number of sources, not the overall r-squared of the fit [40]. Min S/N refers to the smallest estimated signal-to-noise ratio of any of the factors included in the model [40]. A value of Min. R^2^ > 0.80 and a value of Min. S/N > 2.0 usually indicate that the UNMIX model fits well [40]. Table 3 shows the Min. R^2^ and Min. S/N values under different source numbers obtained from the UNMIX model in this study. Obviously, three-factor solution was the optimal choice for UNMIX with the Min. R^2^ value of 0.85 and Min. S/N value of 2.1 (S2 Table). Unlike UNMIX, no strict rules are set to find the optimum number of sources for the PMF model. The best value for the number of sources (*p*) is determined by repeating the analysis with different *p* values [40]. An optimal PMF solution has obtained that had a robust Q value near the theoretical Q value. The theoretical Q value (Q_*t*_) was 1503, which was approximated using the equation Q_*t*_ ≈ *m* × *n*–*p* × (*m* + *n*), where *m* is the number of species and *n* is the number of samples [40]. Consequently, the three-factor solution was chosen. Source profiles for the two models are presented in Fig 2 and details of source composition are listed in Tables 4 and 5. Qualitatively, it apparent that the source profiles identified by the two models showed a high degree of similarity in terms of concentration and percentage (S3 Table; S4 Table).

**Table 3:** Min. R^2^ and Min. S/N values for different source numbers obtained from UNMIX model.

Source numbers	1	2	3	4	5	6	7	8	9	10	11	12
R^2^	0.47	0.75	0.85	0.85	0.91	0.93	0.96	0.97	0.98	0.98	0.99	1.00
S/N	11.79	3.53	2.10	1.93	1.39	1.09	1.00	0.89	0.75	0.62	0.51	0.58

**Table 4:** Source composition (mg kg^-1^) from UNMIX model.

Species	UNMIX_F1	UNMIX_F2	UNMIX_F3	UNMIX_SUM
As	1.460	1.840	6.040	9.340
Cd	0.016	0.145	0.021	0.182
Co	1.260	3.030	8.930	13.22
Cr	8.500	20.500	38.300	67.30
Cu	3.220	8.870	16.800	28.89
Hg	0.068	0.001	0.007	0.076
Mn	79.6	139	382	600.6
Ni	3.430	8.440	19.600	31.47
Pb	3.690	8.030	14.500	26.22
Se	0.017	0.055	0.079	0.151
V	8.150	12.600	56.100	76.85
Zn	11.000	47.300	43.800	102.1
Explained variances (%)	11.7%	24.1%	56.6%	92.4%

**Table 5:** Source composition (mg kg^-1^) from PMF model.

Species	PMF_F1	PMF_F2	PMF_F3	PMF_SUM
As	0.632	5.134	2.040	7.806
Cd	0.008	0.115	0.016	0.139
Co	0.000	6.016	6.034	12.050
Cr	3.861	30.995	27.287	62.143
Cu	0.757	11.321	12.225	24.303
Hg	0.066	0.000	0.008	0.075
Mn	85.850	192.080	362.27	640.200
Ni	0.759	16.200	12.541	29.500
Pb	1.645	11.536	12.040	25.221
Se	0.015	0.057	0.078	0.150
V	3.525	13.102	51.673	68.300
Zn	4.431	40.763	32.236	77.430
Explained variances (%)	9.8%	31.6%	50.1%	91.5%

To further evaluate how well the two methods fit the data, the performance parameters of explained variance (*r*^*2*^) and correlation coefficients for the UNMIX factor profiles and corresponding PMF factor profiles were calculated (Table 6). The results showed that the three-factor solution explained 92.4% and 91.5% of the total variances for UNMIX and PMF, respectively. The minor difference in the r^2^ values might be attributed to the processing methods for noisy analytical data in UNMIX and PMF. Measurement errors were assumed to be identical in UNMIX, whereas PMF considered the noise structure of the analytical data to be heterocedastic. In most of the cases of source apportionment with noisy environmental data, the assumption regarding error structure in UNMIX is unrealistic. In comparison, the uncertainty estimate in PMF plays an important role because it allows each data point to be individually weighed. In this study, most of trace metals in soil samples had relatively high coefficients of variation, indicating a relatively high noise variation. Thus, the higher *r*^*2*^ values may not indicate that UNMIX explained the larger amount of meaningful information than PMF. This might incorporate noise in the UNMIX solution. In general, the resolved solutions by these two models provided sufficient information to represent the majority of the entire dataset. From Table 6, the correlation coefficients between composition (*g*_*k*_) / contribution profiles (*f*_*k*_) from UNMIX and the corresponding profiles from PMF were high in all cases, ranging from 0.848 to 0.999. In particular, the correlation coefficients of composition profiles for each factor obtained from the two models were higher than 0.98. Generally, uncertainty estimation of the analytical data would affect the results of source apportionment, because it reflects the quality and reliability of each measured data point. Different algorithms for the receptor models would also usually produce incompletely consistent factor score matrix and factor loading matrix in the matrix decomposition process. Therefore, the difference of source profiles from UNMIX and PMF might be caused by the uncertainty parameters of the models and their different algorithms. Both models showed good correlation for the same identified factor profiles and produced good results, with minor differences between them, which mutually verified the reliability of the source apportionment.

**Table 6:** Contribution and fits for different factors obtained by UNMIX and PMF.

Factors	Identified sources	*g*_*k*_^a^	*f*_*k*_^b^	SCUNMIX^c^ (%)	SCPMF^d^ (%)
Factor 1	Industrial activities	0.990	0.961	24.9	24.8
Factor 2	Agricultural activities	0.981	0.848	33.3	37.2
Factor 3	Natural source	0.999	0.874	41.8	38.0

^a^: Correlation coefficients between composition profiles from PMF and corresponding profiles from UNMIX

^b^: Correlation coefficients between contribution profiles from PMF and corresponding profiles from UNMIX

^c^: Source contributions by UNMIX

^d^: Source contributions by PMF.

### Source identification

Because similar source profiles for the trace metals in soils of Tianjin were derived from both UNMIX and PMF models (Fig 2), only the PMF solution is further discussed and interpreted here. The spatial distributions of EF and I_*geo*_ were provided as a useful aid to identify hot-spot areas with high levels of contamination and to infer the possible sources of trace metals in soils at the regional scale. Estimated maps of EF and I_*geo*_ in the whole area of Tianjin are presented in S1 Fig and S2 Fig, respectively. A scatter diagram of enrichment factor (EF) for soil trace metals of study area is presented in S3 Fig. The sampling points were plotted in different quadrants indicating that the trace metals were controlled by different sources to varying degree.

The first factor (F1) was characterized by high loading of Hg. As mentioned above, the spatial distribution maps of EF and I_geo_ for Hg indicated that high values were mainly concentrated near the Dagu Wastewater Drainage River. Mercury-containing wastewater generated in industrial activities is discharged into this river for agricultural irrigation, causing an accumulation of Hg in the surrounding soil [39]. Additionally, it has been reported that HgCl has been used as a catalyst for producing polyvinyl chloride by the calcium carbide method in a Dagu chemical plant in recent decades, which has led to increasing Hg emission to the environment [12]. The accumulation of Hg was also reported to be associated with coal combustion in industrial activities [41, 42]. Thus, the factor might represent an anthropogenic source of industry activities from waste emission and coal combustion.

The second factor (F2) was predominantly characterized by high loading of Cd, followed by As, Zn, Co, Cr, Cu and Ni. As analyzed in the previous sections, the calculated results of EF and I_geo_ values revealed that a wide range of agricultural soils in Tianjin were highly contaminated with Cd. The application of chemical fertilizers was usually regarded as an important source of Cd in surface soils [43, 44]. Cadmium can be present in relatively large amounts in phosphate fertilizers ranging from near 0 to more than 150 mg/kg, depending on the provenance of the phosphate rock [45]. Arsenic can be easily emitted into the environment by the application of compound fertilizer and pesticides containing As [46]. Extensively application of fungicides for crops and vegetables is one of the main soil zinc sources in China [46]. In addition to the impact of pesticides and fertilizers, the use of sewage irrigation and sewage sludge is also an important source of trace metals entering agricultural soils [47]. Wastewater from drainage rivers, mainly receiving municipal sewage and industrial wastewater, has been used for irrigation in agricultural areas in Tianjin for decades [48]. Previous studies had shown that heavy metal pollution existed in the wastewater-irrigated area of Tianjin, especially for Cd accumulation [9–10, 39]. From S1 Fig, we identified several hotspots of high Cd and Zn concentration across the study area. Compared to spatial distribution of Cd and Zn, the hotspots of Hg were only distributed near sewage irrigation area. We can distinguish the difference between Cd (or Zn) and Hg in agricultural region far away from the sewage irrigation area. Cumulatively, the sources of Cd and Zn in soil were main originated from agricultural sources, even though the impact of industrial activity on soil Cd and Zn in sewage irrigation area. Together, this factor might indicate an anthropogenic source associated with agricultural activities (e.g., the application of pesticides and fertilizers).

The third factor (F3) was characterized by high loading of V and Mn and slight loading of Co, Cr, Cu, Ni, Pb, Se and Zn. The mean concentrations of V, Mn, Co, Cr, Cu, Ni and Se in soils were below their corresponding background concentrations in Tianjin (Table 1). Approximately 94.7% of I_geo_ values for Co, 98.3% of I_geo_ values for Cr, 91.8% of I_geo_ values for Cu, 95.3% of I_geo_ values for Ni, 97.1% of I_geo_ values for Se and 95.9% of I_geo_ values for V were less than zero (Table 2(B)), indicating that these elements were probably predominantly controlled by natural sources. Previous studies indicated that soil Co, Cr, Mn, Ni and V were highly dependent on soil parent materials [49–51]. Thus, these elements were mainly derived from natural sources. It is worth noting that, the contributions of Co, Cr, Cu and Ni in F2 were similar to their F3. One explanation is that concentrations were a result of varying inputs due to spatial differences in influence factors. For instance, some samples contaminated by agricultural activities were identified as agricultural sources. While, some samples with higher concentrations for Co, Cr, Cu and Ni were controlled by the parent material. From the study area as a whole, Co, Cr, Cu and Ni in soil across the study area originated from both natural and agricultural sources.

To test the potential mixing relationships among mixing endmembers. Correlation analysis were done by using commercial statistics software package SPSS for Windows (SPSS Inc. Quarry Bay, HK). Tables 7 and 8 presents pearson correlation coefficients and their significance levels. The natural sources exhibited significantly negative correlations (p<0.01) with industrial sources in PMF (r = -0.243**) and UNMIX (r = -0.517**). The natural sources also exhibited significantly negative correlations (p<0.01) with agriculture sources in PMF (r = -0.410**) and UNMIX (r = -0.518**). These results indicate the natural source was much different from anthropogenic sources (agricultural sources and/or industrial sources). This conclusion confirmed the third factor (F3) was mainly derived from natural source, whereas the first factor (F1) and second factor (F2) were derived from anthropogenic sources.

**Table 7:** Pearson correlation coefficients of different sources obtained from UNMIX model.

	Factor 1	Factor 2	Factor3
Factor 1	1.000	-0.007	-0.517**
Factor 2		1.000	-0.518**
Factor3			1.000

** Correlation is significant at P < 0.01 (two-tailed).

Factor 1: Industrial activities; Factor 2: Agricultural activities; Factor 3: Natural source.

**Table 8:** Pearson correlation coefficients of different sources obtained from PMF model.

	Factor 1	Factor 2	Factor 3
Factor 1	1.000	0.057	-0.243**
Factor 2		1.000	-0.410**
Factor 3			1.000

** Correlation is significant at P < 0.01 (two-tailed).

Factor 1: Industrial activities; Factor 2: Agricultural activities; Factor 3: Natural source.

### Source contributions

The percent contribution from each source was calculated based on the average of the individual percent contributions and the results are presented in Table 6. The first common source was mainly generated by coal combustion and waste emission in industrial activities, with relative contributions of 24.9% for UNMIX and 24.8% for PMF. The secondary source (agricultural activities) identified by UNMIX and PMF models, which was mainly associated with the application of pesticides and fertilizers and sewage irrigation, accounted for 33.3% and 37.2%, respectively, of the total contributions. Natural sources were responsible for the most significant contribution extracted by UNMIX (41.8%) and PMF (38.0%) models. Both models provided virtually the same contributions and similar spatial distributions for all the explained sources. In addition, both UNMIX and PMF models have their special features with different algorithms and parameter settings. Therefore, it is essential to use multiple methods to estimate potential sources and contributions to confirm the validity of source apportionment.

**Fig 3:**
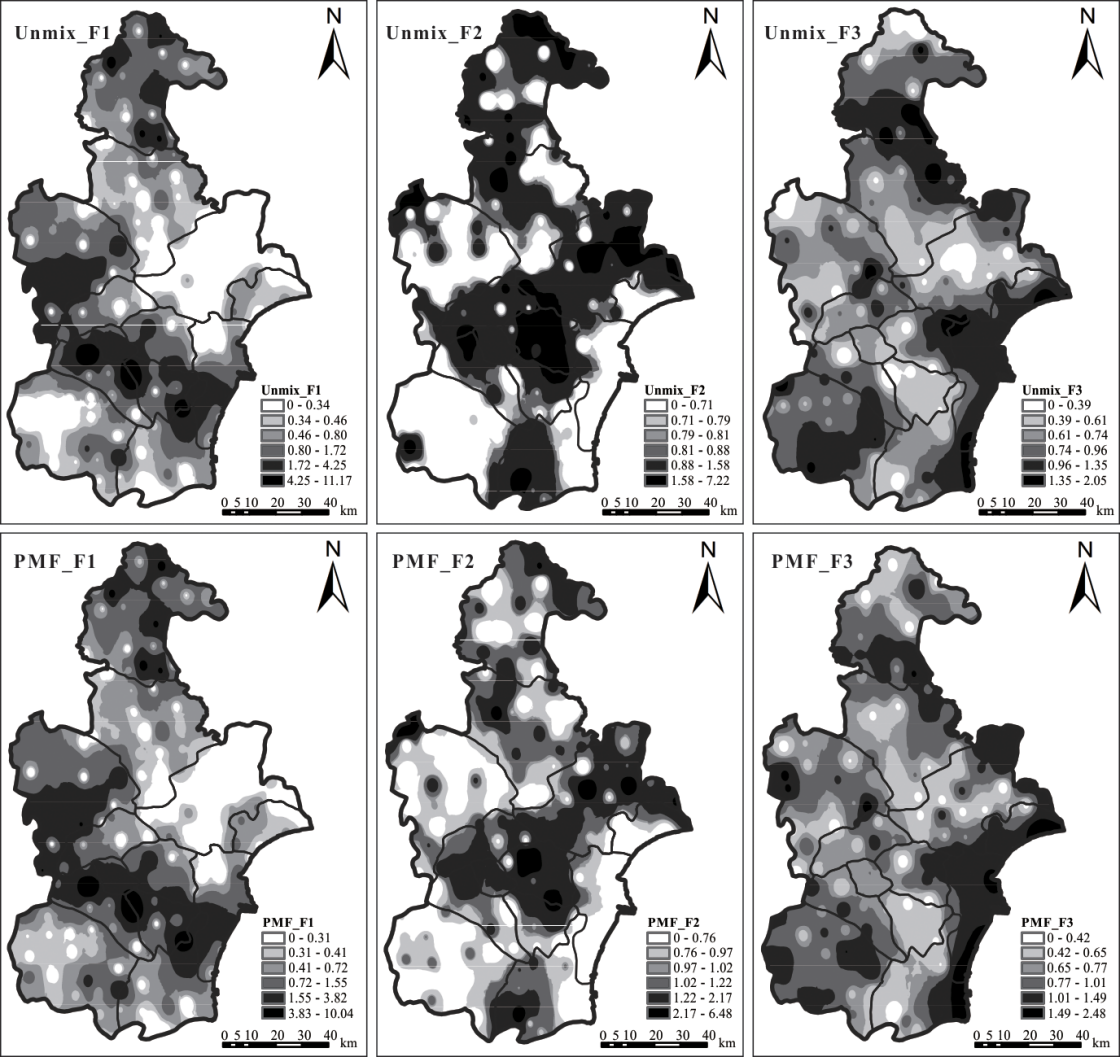
Spatial distribution of factor scores calculated from UNMIX (Upper) and PMF (Below) across study area.

The spatial variation of different source contributions obtained from UNMIX and PMF results was shown in Fig 3, clearly showing the respective regions that were affected by the three sources. The spatial distribution maps of F1 that were identified by the two models showed almost identical results. The main area under the influence of this factor was in the region with high concentrations of Hg. The affected regions of F2 and F3 identified by the two models showed similar results with minor differences. High values of F2 were concentrated in the urban and suburban areas with dense population and frequent agricultural activities. The distributions of F3 demonstrated that most of the high values were located in the outer suburbs and coastal areas less affected by human activities. The apportionment results suggested that the impact of human activities on trace metals in soils of the Tianjin area cannot be ignored because of the rapid development over past decades.

## Conclusions

In this study, EF, Igeo, PMF, and UNMIX, combined with a GIS tool, were used to identify contamination characteristics and provide a quantitative source apportionment of soil trace metals in Tianjin. The results showed that Cd was the most significant pollutant that covered a wide range of contaminated areas. Low levels of pollution by Hg, Pb and Zn were distributed in certain regions of Tianjin. The other measured metals (As, Co, Cr, Cu, Mn, Ni, Se and V) showed no or minimal contamination and were sporadically distributed across the study area. Industrial activities, agricultural activities and natural sources were apportioned as potential sources, with relative contributions of 24.8–24.9%, 33.3–37.2% and 38.0–41.8% for soil trace metals, respectively. Spatially, the regions under the influence of industrial activities and agricultural activities mainly occurred in the urban and suburban areas of Tianjin, whereas the outer suburbs and coastal areas were less affected by human activities. In addition, both the UNMIX and PMF models produced comparable results with good agreement between respective composition and contribution profiles among the three factors, mutually confirming the source apportionment. Therefore, the results of this study demonstrate that the combination of the contamination index, geographic information systems, and multivariate receptor models, which account for spatial variability, can be a good approach to use for the source apportionment of soil trace metals on a regional scale.

The application of these models in soil trace metals has not been well studied to date, and it can be difficult to test because of the complexities of soil geochemistry [51, 52]. Recently, some researchers have successfully used PMF and UNMIX to trace the source of heavy metals in soils [53–59]. Most of their study areas were of limited size, having similar geology and rainfall, thereby providing a relatively homogenous site for the application of these models. Chen at al. performed a source apportionment for surface soils of the Beijing metropolitan area, which was an exception [60]. The receptor model assumes, in theory, that the concentration of a chemical element is the linear summation of the contributors, with no reaction between the emissions from different sources and any formation or elimination of the substance during transmission. The modeling errors were caused by using a model in which the true physical-chemical phenomena were simplified. The instability of a receptor model due to nearly collinear sources is often worsened by a large number of unknown sources. When a study area becomes larger, the variability of the values increases, and the pollutants from unknown sources increase [61]. The similarity of these source profiles usually affects the results of the receptor models. If such data are apportioned by the PMF model or the UNMIX model, one factor extracted may contain these nearly collinear sources. There have been concerns that using PMF and UNMIX alone for source apportionment cannot reliably identify and apportion pollution sources, as it ignores the geochemical relevance such as pollutants from the sources that usually do not migrate to the entire area.

Increasing the number of fitting species and samples would enhance the accuracy of the model outcome. However, doing so is very costly and often prohibited by the available resources. Thus, simplifying the assumption of sources and contamination pathways in soil is inevitable but provides a potential application of the receptor model in source apportionment. In view of this study of source apportionment for soil trace metals in Tianjin, the results still show relatively large error. Therefore, the application of multivariate receptor modeling to apportioning sources of soil heavy metals at a regional scale remains a challenge.

## Supporting information

S1 Fig Spatial distribution maps of enrichment factor (EF) for soil trace metals of study area. (TIF)

S2 Fig Spatial distribution maps of geoaccumulation index (I_*geo*_) for soil trace metals of study area. (TIF)

S3 Fig Scatter diagram of enrichment factor (EF) for soil trace metals of study area. (TIF)

S1 Table Percentages of class distribution for pollution assessment of trace metals in soil samples using enrichment factor index (a) and geoaccumulation index (b).
(DOCX)

S2 Table Min. R^2^ and Min. S/N values for different source numbers obtained from UNMIX model. (DOCX)

S3 Table Source composition (mg kg^-1^) from UNMIX model. (DOCX)

S4 Table Source composition (mg kg^-1^) from PMF model. (DOCX)

S5 Table Details of input data from UNMIX and PMF models. (DOCX)
