# Supplementary figures and images for: Application of a combined approach including contamination indexes, geographic information system and multivariate statistical models in levels, distribution and sources study of metals in soils in Northern China
Source: PLoS One. 2018 Feb 23;13(2):e0190906. doi: 10.1371/journal.pone.0190906 (PMC5825019; doi:10.1371/journal.pone.0190906)

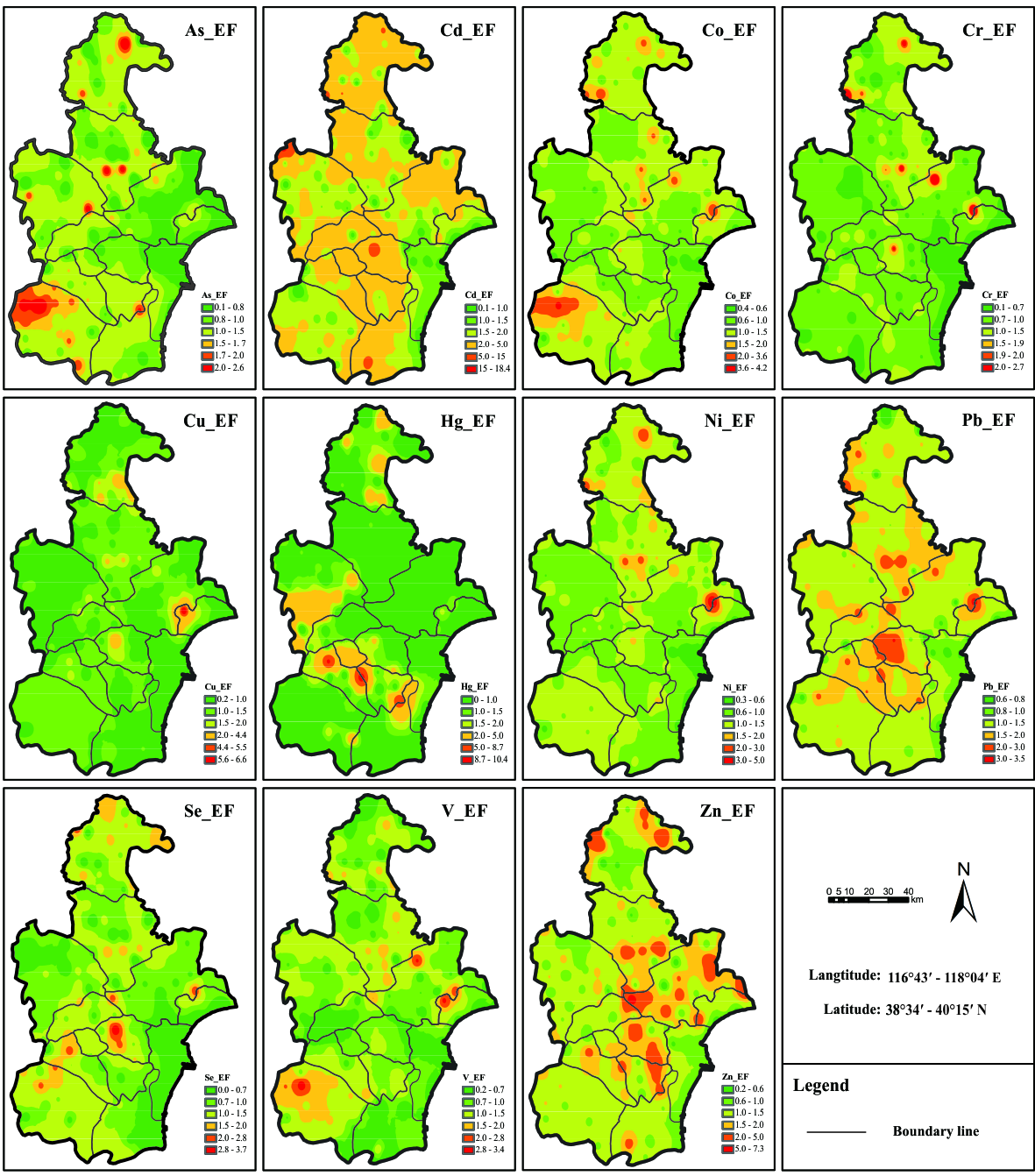

Supplement: S1 Fig — (TIF) [file pone.0190906.s001.tif]

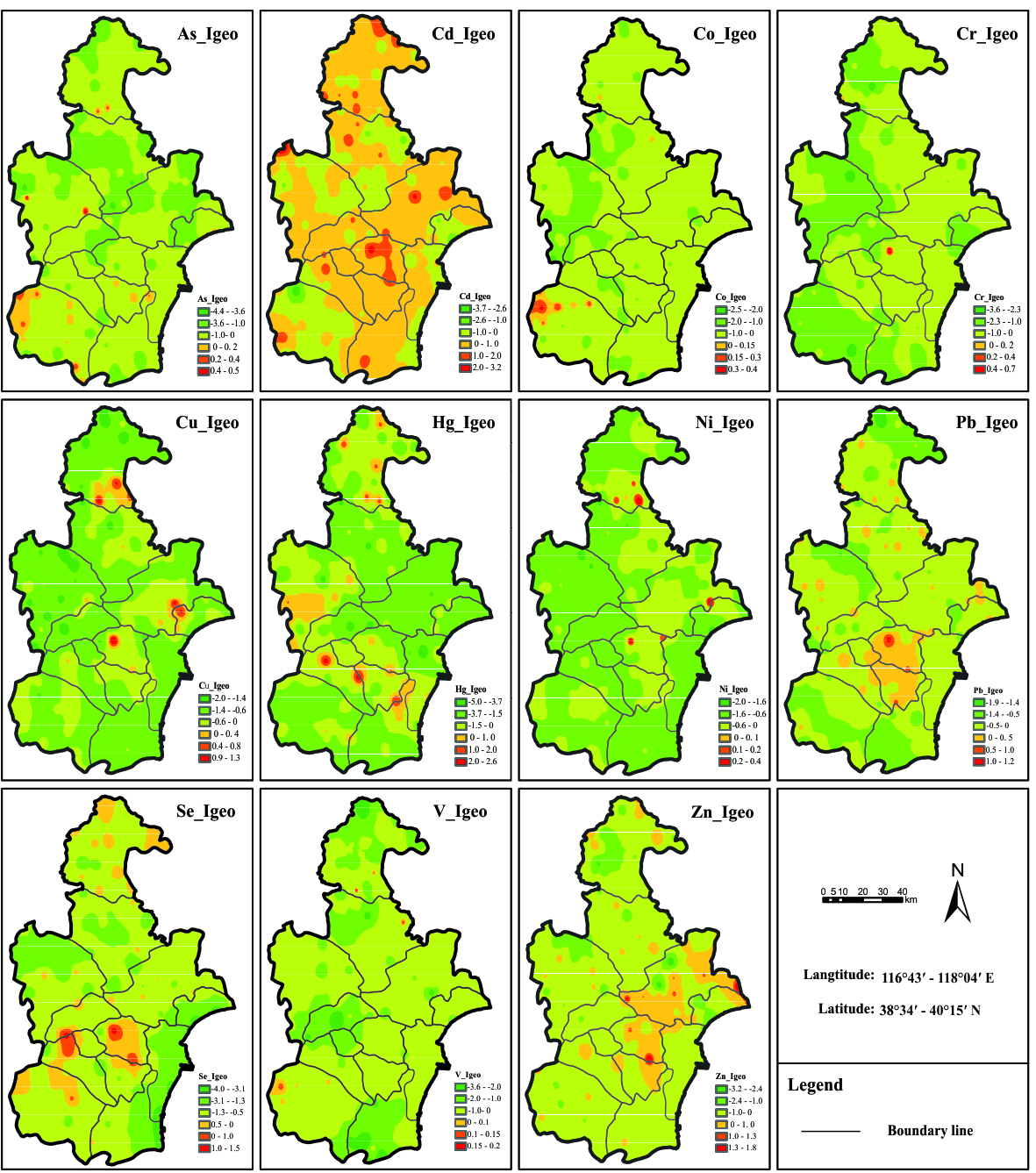

Supplement: S2 Fig — (TIF) [file pone.0190906.s002.tif]

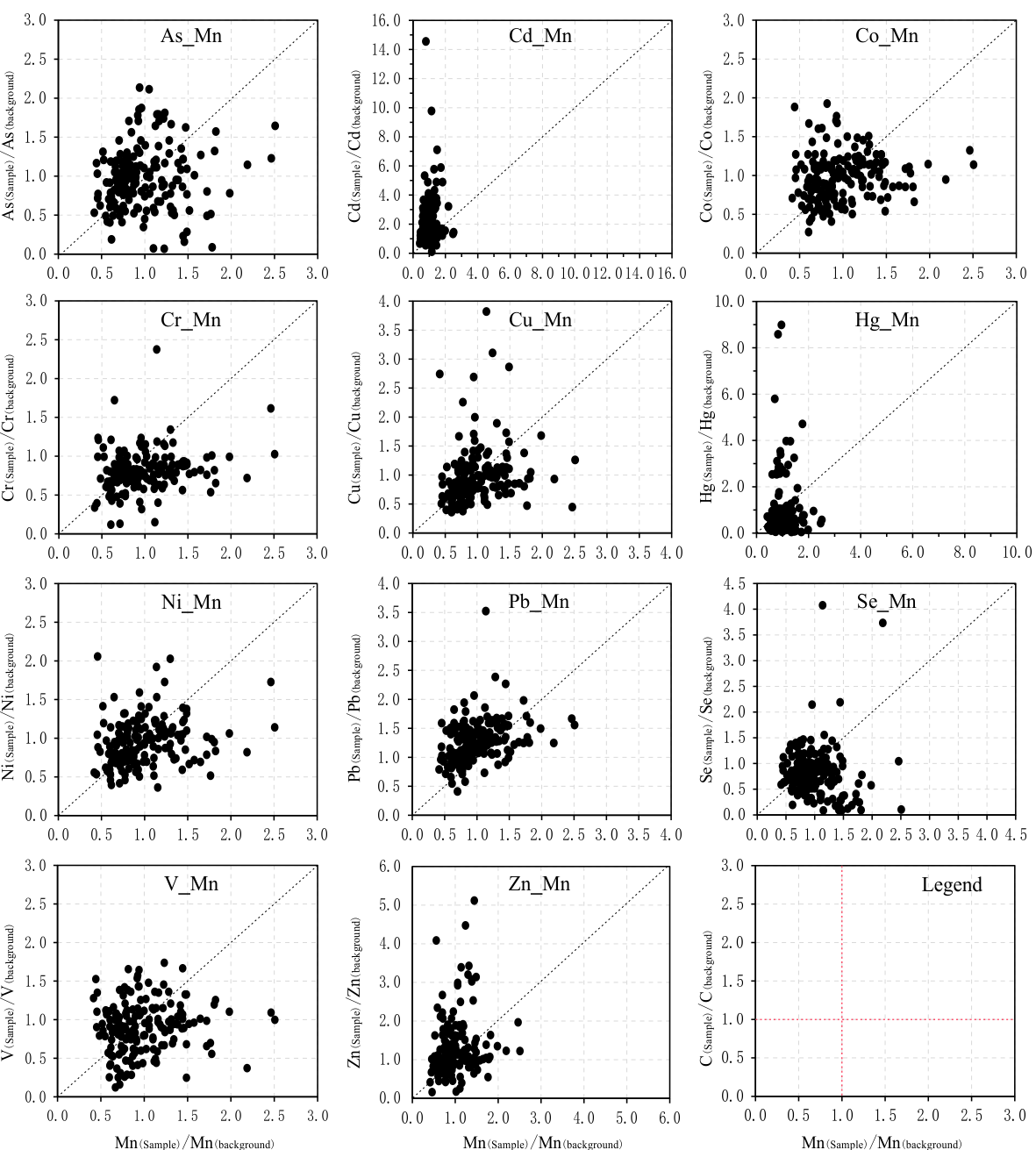

Supplement: S3 Fig — (TIF) [file pone.0190906.s003.tif]
