# Supplementary material for: Application of a combined approach including contamination indexes, geographic information system and multivariate statistical models in levels, distribution and sources study of metals in soils in Northern China
Source: PLoS One. 2018 Feb 23;13(2):e0190906. doi: 10.1371/journal.pone.0190906 (PMC5825019; doi:10.1371/journal.pone.0190906)
Supplement: S1 Table — Percentages of class distribution for pollution assessment of trace metals in soil samples using enrichment factor index (a) and geoaccumulation index (b). (DOCX) [file pone.0190906.s004.docx]

**S1 Table.** percentages of class distribution for pollution assessment of trace metals in soil samples using enrichment factor index (a) and geoaccumulation index (b)

| Trace metals | As | Cd | Co | Cr | Cu | Hg | Ni | Pb | Se | V | Zn |
| --- | --- | --- | --- | --- | --- | --- | --- | --- | --- | --- | --- |
| (a) |  |  |  |  |  |  |  |  |  |  |  |
| no or minimal enrichment | 95.3 | 52.1 | 93.0 | 96.5 | 94.7 | 80.3 | 97.1 | 83.8 | 95.3 | 94.9 | 78.0 |
| moderate enrichment | 4.7 | 44.4 | 6.4 | 3.5 | 4.7 | 17.1 | 2.9 | 16.2 | 4.7 | 5.1 | 21.4 |
| significant enrichment | 0.0 | 3.5 | 0.6 | 0.0 | 0.6 | 2.6 | 0.0 | 0.0 | 0.0 | 0.0 | 0.6 |
| (b) |  |  |  |  |  |  |  |  |  |  |  |
| uncontaminated | 89.5 | 41.5 | 94.7 | 98.3 | 91.8 | 85.4 | 95.3 | 77.2 | 97.1 | 95.9 | 74.9 |
| uncontaminated to moderately contaminated | 10.5 | 45.6 | 5.3 | 1.8 | 7.0 | 8.8 | 4.7 | 22.2 | 1.8 | 4.1 | 19.9 |
| moderately contaminated | 0.0 | 11.1 | 0.0 | 0.0 | 1.2 | 4.7 | 0.0 | 0.6 | 1.2 | 0.0 | 5.3 |
| moderately to heavily contamined | 0.0 | 1.2 | 0.0 | 0.0 | 0.0 | 1.2 | 0.0 | 0.0 | 0.0 | 0.0 | 0.0 |
| heavily contaminated | 0.0 | 0.6 | 0.0 | 0.0 | 0.0 | 0.0 | 0.0 | 0.0 | 0.0 | 0.0 | 0.0 |
