# Supplementary material for: Application of a combined approach including contamination indexes, geographic information system and multivariate statistical models in levels, distribution and sources study of metals in soils in Northern China
Source: PLoS One. 2018 Feb 23;13(2):e0190906. doi: 10.1371/journal.pone.0190906 (PMC5825019; doi:10.1371/journal.pone.0190906)
Supplement: S2 Table — (DOCX) [file pone.0190906.s005.docx]

**S2 Table.** Min. R^2^ and Min. S/N values for different source numbers obtained from UNMIX model

| Source numbers | 1 | 2 | 3 | 4 | 5 | 6 | 7 | 8 | 9 | 10 | 11 | 12 |
| --- | --- | --- | --- | --- | --- | --- | --- | --- | --- | --- | --- | --- |
| R^2^ | 0.47 | 0.75 | 0.85 | 0.85 | 0.91 | 0.93 | 0.96 | 0.97 | 0.98 | 0.98 | 0.99 | 1.00 |
| S/N | 11.79 | 3.53 | 2.10 | 1.93 | 1.39 | 1.09 | 1.00 | 0.89 | 0.75 | 0.62 | 0.51 | 0.58 |
