# Supplementary material for: Application of a combined approach including contamination indexes, geographic information system and multivariate statistical models in levels, distribution and sources study of metals in soils in Northern China
Source: PLoS One. 2018 Feb 23;13(2):e0190906. doi: 10.1371/journal.pone.0190906 (PMC5825019; doi:10.1371/journal.pone.0190906)
Supplement: S3 Table — (DOCX) [file pone.0190906.s006.docx]

**S3 Table.** Source composition (mg kg^-1^) from UNMIX model

| Species | UNMIX_F1 | UNMIX_F2 | UNMIX_F3 | UNMIX_SUM |
| --- | --- | --- | --- | --- |
| As | 1.460 | 1.840 | 6.040 | 9.340 |
| Cd | 0.016 | 0.145 | 0.021 | 0.182 |
| Co | 1.260 | 3.030 | 8.930 | 13.22 |
| Cr | 8.500 | 20.500 | 38.300 | 67.30 |
| Cu | 3.220 | 8.870 | 16.800 | 28.89 |
| Hg | 0.068 | 0.001 | 0.007 | 0.076 |
| Mn | 79.6 | 139 | 382 | 600.6 |
| Ni | 3.430 | 8.440 | 19.600 | 31.47 |
| Pb | 3.690 | 8.030 | 14.500 | 26.22 |
| Se | 0.017 | 0.055 | 0.079 | 0.151 |
| V | 8.150 | 12.600 | 56.100 | 76.85 |
| Zn | 11.000 | 47.300 | 43.800 | 102.1 |
| Explained variances (%) | 11.7% | 24.1% | 56.6% | 92.4% |
