# Supplementary material for: Application of a combined approach including contamination indexes, geographic information system and multivariate statistical models in levels, distribution and sources study of metals in soils in Northern China
Source: PLoS One. 2018 Feb 23;13(2):e0190906. doi: 10.1371/journal.pone.0190906 (PMC5825019; doi:10.1371/journal.pone.0190906)
Supplement: S4 Table — (DOCX) [file pone.0190906.s007.docx]

**S4 Table.** Source composition (mg kg^-1^) from PMF model

| Species | PMF_F1 | PMF_F2 | PMF_F3 | PMF_SUM |
| --- | --- | --- | --- | --- |
| As | 0.632 | 5.134 | 2.040 | 7.806 |
| Cd | 0.008 | 0.115 | 0.016 | 0.139 |
| Co | 0.000 | 6.016 | 6.034 | 12.050 |
| Cr | 3.861 | 30.995 | 27.287 | 62.143 |
| Cu | 0.757 | 11.321 | 12.225 | 24.303 |
| Hg | 0.066 | 0.000 | 0.008 | 0.075 |
| Mn | 85.850 | 192.080 | 362.27 | 640.200 |
| Ni | 0.759 | 16.200 | 12.541 | 29.500 |
| Pb | 1.645 | 11.536 | 12.040 | 25.221 |
| Se | 0.015 | 0.057 | 0.078 | 0.150 |
| V | 3.525 | 13.102 | 51.673 | 68.300 |
| Zn | 4.431 | 40.763 | 32.236 | 77.430 |
| Explained variances (%) | 9.8% | 31.6% | 50.1% | 91.5% |
