# Supplementary material for: Application of a combined approach including contamination indexes, geographic information system and multivariate statistical models in levels, distribution and sources study of metals in soils in Northern China
Source: PLoS One. 2018 Feb 23;13(2):e0190906. doi: 10.1371/journal.pone.0190906 (PMC5825019; doi:10.1371/journal.pone.0190906)
Supplement: S5 Table — (DOCX) [file pone.0190906.s008.docx]

**S5 Table.** Details of input data from UNMIX and PMF models (mg/kg)

| Species | Min | 10th | 25th | 50th | Mean | 75th | 90th | Max |
| --- | --- | --- | --- | --- | --- | --- | --- | --- |
| As | 0.67 | 4.85 | 6.59 | 9.17 | 9.34 | 11.4 | 15.08 | 20.5 |
| Cd | 0.01 | 0.09 | 0.11 | 0.15 | 0.183 | 0.2 | 0.318 | 1.31 |
| Co | 3.7 | 7.8 | 10 | 13.2 | 13.3 | 15.6 | 18.5 | 26.2 |
| Cr | 10 | 45.6 | 57.5 | 66.5 | 67.9 | 76.2 | 90 | 200 |
| Cu | 10.4 | 15.2 | 20 | 25 | 28.1 | 32.3 | 40.7 | 110 |
| Hg | 0.004 | 0.015 | 0.022 | 0.037 | 0.076 | 0.078 | 0.218 | 0.755 |
| Mn | 285 | 414 | 492 | 615 | 681 | 825 | 1008 | 1720 |
| Ni | 12.1 | 19.7 | 24.4 | 30.8 | 31.4 | 37 | 43.7 | 68.5 |
| Pb | 8.6 | 18 | 21.5 | 25.8 | 26.4 | 30.9 | 34.4 | 74 |
| Se | 0.016 | 0.051 | 0.102 | 0.145 | 0.149 | 0.181 | 0.23 | 0.734 |
| V | 10.4 | 37.9 | 57 | 77 | 76.8 | 94 | 115.8 | 148 |
| Zn | 12.8 | 52.3 | 65.5 | 82.5 | 101 | 119 | 174.4 | 406 |
